# Prebiotic inulin enhances gut microbial metabolism and anti-inflammation in apolipoprotein E4 mice with sex-specific implications

**DOI:** 10.1038/s41598-023-42381-x

**Published:** 2023-09-13

**Authors:** Ya-Hsuan Chang, Lucille M. Yanckello, George E. Chlipala, Stefan J. Green, Chetan Aware, Amelia Runge, Xin Xing, Anna Chen, Kathryn Wenger, Abeoseh Flemister, Caixia Wan, Ai-Ling Lin

**Affiliations:** 1 Department of Pharmacology and Nutritional Sciences, University of Kentucky, Lexington, KY 40536 USA; 2 Sanders Brown Center on Aging, University of Kentucky, Lexington, KY 40536 USA; 3 Department of Radiology, University of Missouri, Columbia, MO 65212 USA; 4 NextGen Precision Health, University of Missouri, Columbia, MO 65212 USA; 5 Research Informatics Core, University of Illinois Chicago, Chicago, IL 60612 USA; 6 Genomics and Microbiome Core Facility, Rush University, Chicago, IL 60612 USA; 7 Department of Biological Sciences, University of Missouri, Columbia, MO 65211 USA; 8 Department of Computer Science, University of Kentucky, Lexington, KY 40506 USA; 9 Department of Biochemistry, University of Missouri, Columbia, MO 65211 USA; 10 Department of Biological and Biomedical Engineering, University of Missouri, Columbia, MO 65211 USA; 11 Institute for Data Science and Informatics, University of Missouri, Columbia, MO 65211 USA

**Keywords:** Microbiology, Medical research

## Abstract

Gut dysbiosis has been identified as a crucial factor of Alzheimer's disease (AD) development for apolipoprotein E4 (*APOE4*) carriers. Inulin has shown the potential to mitigate dysbiosis. However, it remains unclear whether the dietary response varies depending on sex. In the study, we fed 4-month-old *APOE*4 mice with inulin for 16 weeks and performed shotgun metagenomic sequencing to determine changes in microbiome diversity, taxonomy, and functional gene pathways. We also formed the same experiments with *APOE*3 mice to identify whether there are *APOE*-genotype dependent responses to inulin. We found that *APOE4* female mice fed with inulin had restored alpha diversity, significantly reduced *Escherichia coli* and inflammation-associated pathway responses. However, compared with *APOE4* male mice, they had less metabolic responses, including the levels of short-chain fatty acids-producing bacteria and the associated kinases, especially those related to acetate and *Erysipelotrichaceae*. These diet- and sex- effects were less pronounced in the *APOE3* mice, indicating that different *APOE* variants also play a significant role. The findings provide insights into the higher susceptibility of *APOE4* females to AD, potentially due to inefficient energy production, and imply the importance of considering precision nutrition for mitigating dysbiosis and AD risk in the future.

## Introduction

Apolipoprotein E4 (*APOE4*) is the strongest genetic risk factor for Alzheimer's disease (AD), the most common form of dementia characterized by extracellular beta-amyloid plaques, intraneuronal tau tangles, and neurodegeneration^1,2^. Recent evidence suggests that an imbalanced gut microbiome, or dysbiosis, may contribute to developing AD-like neuropathology^3–5^. Specifically, AD patients have an increased abundance of *Escherichia coli (E. coli)*, decreased short-chain fatty acids (SCFAs) as assessed through fecal sampling, and increased systemic inflammation and microglia activation^3^. In addition to the *APOE4* genetic factor, gender plays a significant role with females having a higher risk of AD than males^6–8^. Studies have found that *APOE* alleles and sex influence the gut microbiome structure^9–11^. Asymptomatic *APOE4* carriers have more extensive gut dysbiosis than non-carriers, such as those with *APOE* e3 alleles (*APOE3)*^12^. In addition, females may have different gut microbiota composition than males, increasing the risk for AD pathology^8^. Thus, mitigating dysbiosis at an early stage may be crucial for preventing AD development.

Our prior research demonstrates that supplementing a diet with the prebiotic inulin, a fermentable prebiotic fiber, can positively impact gut microbiome composition, boost the production of SCFAs, enhance mitochondrial function, and decrease neuroinflammation in young, asymptomatic *APOE4* mice^13^. Our results also demonstrate that these effects of dietary inulin supplementation revealed an *APOE* genotype-dependent response^9^. Despite these promising results, it remains unclear whether the response to the inulin diet varies based on sex and whether specific microbial metabolic pathways would be impacted. The present study aims to address this knowledge gap.

We had young, asymptomatic *APOE4* male (*E4*-M) and female (*E4*-F) mice fed with either inulin or control diet for 16 weeks and collected their fecal samples pre and post the diet. We performed shotgun metagenomics sequencing and analyses on microbiome diversity, differential relative abundance of microbial taxonomy, and the Kyoto Encyclopedia of Genes and Genomes (KEGG) functional pathways. We found that *E4*-F and *E4*-M mice had distinctive responses to the diet. *E4*-F fed with inulin (*E4*-F-inulin) had altered β-diversity, and the impacts on the functional pathway were more on anti-inflammation, with significantly decreased *E. coli* abundance compared with the *E4*-F-control group. In addition, inulin normalized alpha (α)-diversity of *E4*-F mice compared with *E4*-M-inulin and *E4*-M-control mice, indicating restoration of evenness and richness of microbial community^14^ of the *E4*-F-inulin mice. Similarly, *E4*-M mice fed with inulin (*E4*-M-inulin) had altered beta (β)-diversity but had significantly increased SCFAs-producing bacteria, especially those related to acetate production, and decreased lactic acid bacteria (LAB). They also had increased gut abundance of SCFAs-related kinases, including acetate CoA-transferase, propionate kinase, and butyrate kinase, and family *Erysipelotrichaceae* contributed most to the abundance of acetate CoA-transferase and propionate kinase. The mitochondrial tricarboxylic acid (TCA) cycle level was also elevated in the *E4*-M-inulin mice.

We performed the same experiments with mice having the human *APOE3* allele. Although inulin altered β-diversity in both sexes, and the *E3*-M-inulin mice also had positive responses on SCFAs-producing bacteria and TCA cycle elevation as the *E4*-M mice did, the overall sex effects were not as strong as those observed in the *APOE4* groups. The findings indicate a significant role played by different APOE variants.

These results highlight the importance of both *APOE* genotype and sex on the gut microbiome changes in response to the inulin. In particular, sex difference plays a significant role in the *APOE4* mice. The findings may shed light on the importance of considering precision nutrition for mitigating *APOE4*-related neurodegenerative disorders, such as AD.

## Results

### Inulin's effects on food intake and body weight in *APOE3 *and *APOE4* Mice

**Figure 1:**
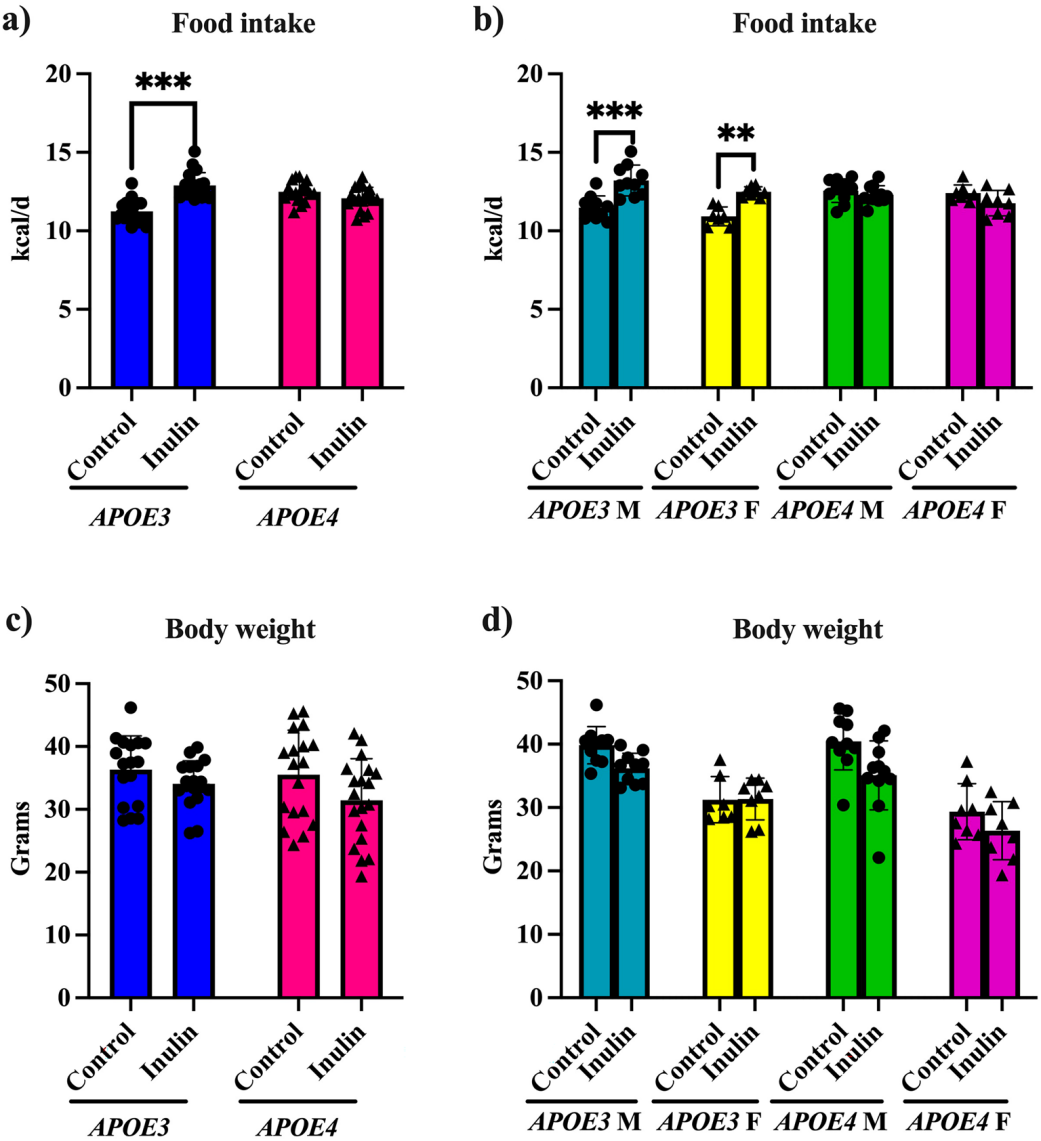
Analysis of food intake and body weight. (**a**) Inulin increased the food intake in *APOE3* mice compared to its control when stratifying mice by *APOE* genotype and diet. (**b**) When stratifying mice by genotype, sex, and diet, inulin increased the food intake in *E3*-M and *E3*-F mice compared to their controls. Inulin didn’t change the body weight of the mice when considering (**c**) genotype and diet or (**d**) genotype, diet, and sex. *p < 0.05. ***p < 0.001.

We documented food intake and body weight changes throughout the study. Figure 1 shows the end-point results. Food intakes were increased in the *APOE3* mice (p < 0.001) (Fig. 1a), both in the male and female mice (Fig. 1b). In contrast, *APOE*4 mice did not show changes in food intake. However, the food intake increases in the *APOE*3 mice did not alter the body weight (Fig. 1c,d). No body weight changes were observed in the *APOE*4 mice either.

### Inulin normalizes α-diversity of the *APOE4* female mice

We analyzed the α-diversity using the Shannon index, which measures the richness or evenness of a microorganism within a sample^14^. Table 1 shows the changes in α-diversity caused by inulin in male and female mice with *APOE3* and *APOE4* genes (considering the interaction of Gene × Sex × Diet), reporting data from a generalized linear model (GLM) analysis and pairwise comparisons, including degrees of freedom, residual df, F value, and p-values.

**Table 1:** α**-**diversity changed by inulin in *APOE3* and *APOE4* male and female mice (gene × sex × diet).

GLM					Pairwise comparisons		
Term	df	Residual df	F	P	Group comparisons	P	
Sex	1	69	6.753	0.012*	*E3*-M-Inulin vs. *E4*-M-Inulin	0.809	
					*E3*-M-Inulin vs. *E3*-F-Inulin	0.203	
					*E3*-M-Inulin vs. *E3*-M-Control	0.166	
					*E4*-M-Inulin vs. *E4*-F-Inulin	0.129	
Diet	1	68	1.514	0.223	*E4*-M-Inulin vs. *E4*-M-Control	0.512	
					*E3*-F-Inulin vs. *E4*-F-Inulin	0.382	
					*E3*-F-Inulin vs. *E3*-F-Control	0.613	
					*E4*-F-Inulin vs. *E4*-F-Control	0.105	
Genotype	1	70	0.307	0.581	*E3*-M-Control vs. *E4*-M-Control	0.579	
					*E3*-M-Control vs. *E3*-F-Control	0.088	
					*E4*-M-Control vs. *E4*-F-Control	0.027*	
					*E3*-F-Control vs. *E4*-F-Control	0.002**	

ADONIS						Pairwise ANOSIM	
Factor	Sum of Square	Mean Square	F	R^2^	P	Group A vs. Group B	P
Diet	0.470	0.470	57.873	0.436	0.001**	*E3*-Control vs. *E3*-Inulin	0.001**
						*E3*-Control vs. *E4*-Control	0.010*
Genotype	0.038	0.038	4.630	0.0348	0.008**	*E3*-Inulin vs. *E4*-Inulin	0.001**
						*E4*-Control vs. *E4*-Inulin	0.001**
Sex	0.022	0.022	2.817	0.020	0.036*	*E3*-F-Control vs. *E3*-F-Inulin	0.003**
						*E3*-F-Control vs. *E3*-M-Control	0.408
						*E3*-F-Control vs. *E4*-F-Control	0.101
						*E3*-F-Inulin vs. *E3*-M-Inulin	0.020*
Diet	0.470	0.470	60.175	0.436	0.001**	*E3*-F-Inulin vs. *E4*-F-Inulin	0.009**
						*E3*-M-Control vs. *E3*-M-Inulin	0.001**
						*E3*-M-Control vs. *E4*-M-Control	0.027*
						*E3*-M-Inulin vs. *E4*-M-Inulin	0.001**
Genotype	0.038	0.038	4.814	0.035	0.004**	*E4*-F-Control vs. *E4*-F-Inulin	0.001**
						*E4*-F-Control vs. *E4*-M-Control	0.004**
						*E4*-F-Inulin vs. *E4*-M-Inulin	0.133
						*E4*-M-Control vs. *E4*-M-Inulin	0.001**

In generalized linear model (GLM) analysis, degrees of freedom (df), residual df, F value, and p-value in the effects of sex, diet, and genotype were reported. In pairwise comparisons, the p-value in each comparison of the means of two groups were reported. β-diversity changed by inulin in *APOE4* and *APOE3* mice (gene × diet) and in *APOE3* and *APOE4* male and female mice (gene × sex × diet). In ADONIS analysis, the sum of square, mean square, F value, R^2^, and p-value in the effects of diet and genotype and sex, diet, and genotype were reported, respectively. In pairwise ANOSIM comparisons, the p-value in each comparison of two groups were reported. *p < 0.05. **p < 0.01.

**Figure 2:**
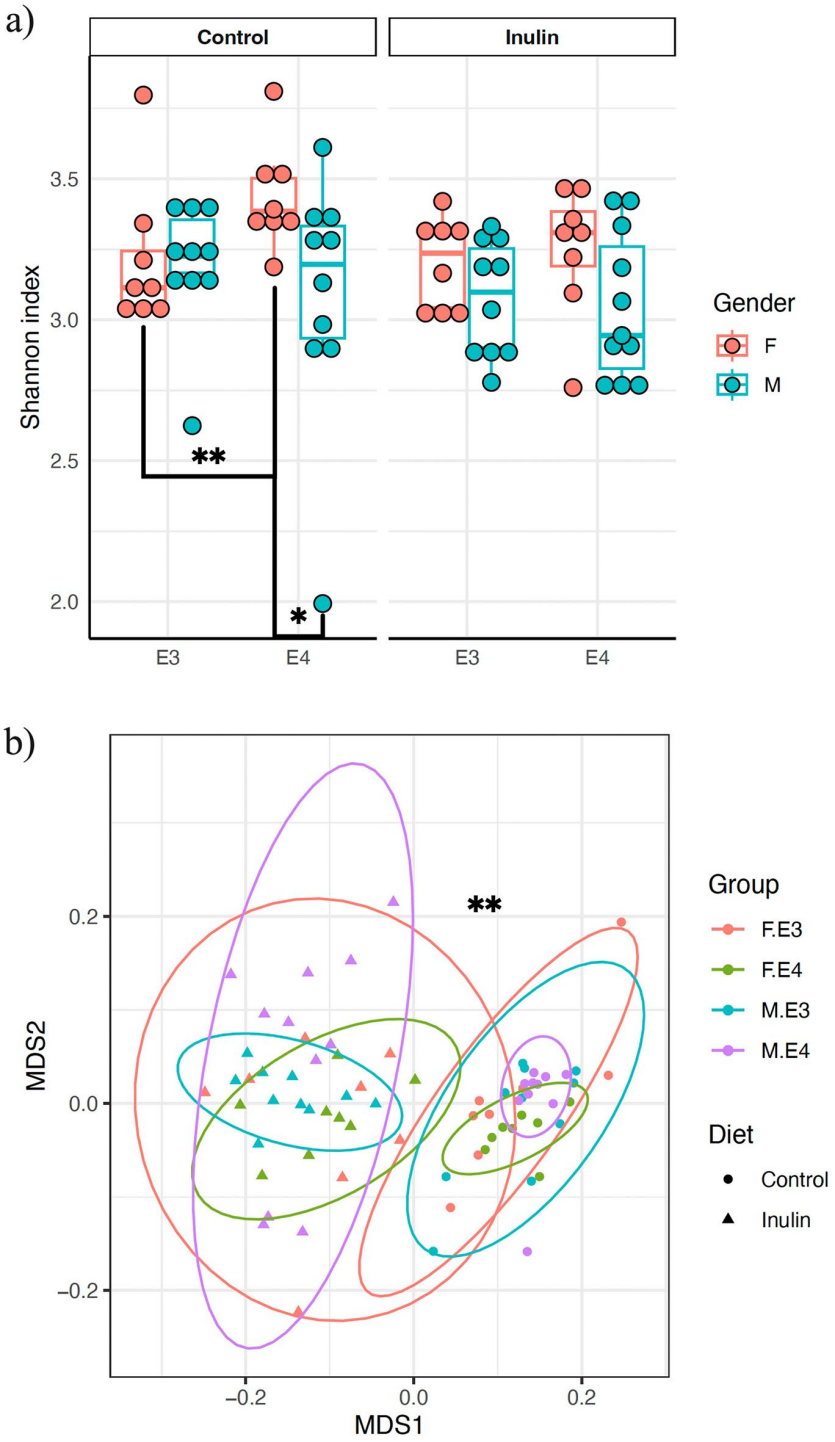
Differences in α-diversity when stratifying mice by *APOE* genotype, sex, and diet and differences in β-diversity when stratifying mice by genotype, sex, and diet. (**a**) Inulin normalized the difference in the α-diversity between *E4*-F-control mice compared to *E3*-F-control and *E4*-M-control mice. However, there was no difference between *E4*-F-inulin, *E3*-F-inulin, and *E4*-M-inulin mice, indicating that inulin normalized the gut dysbacteriosis in *E4*-F mice. Inulin altered the β-diversity in (**b**) *E3*-inulin and *E4*-inulin mice compared to their controls in male and female mice. *p < 0.05. **p < 0.01.

The results are visualized in Fig. 2a. It shows that in the control groups, the α-diversity of *E4*-F was higher than that in *E4*-M (p = 0.027) and *APOE3* female control (*E3*-F-control) mice (p = 0.002) with an overall sex effect among groups [F (1, 69) = 6.753, p = 0.012]; there were no differences between the *E4*-M or *E3*-M mice, either with the control or inulin diets. However, when the mice were given an inulin diet, the difference between *E4*-F and the other three groups diminished, indicating that the diet normalized the α-diversity of the *E4*-F mice.

### Inulin alters β-diversity in both sexes

We analyzed the β-diversity using the Bray–Curtis index, which measures distinct differences in microbial composition between the control mice and those fed with inulin in our study^14^. Table 1 shows the data of changes in β-diversity due to inulin in *APOE3* and *APOE4* mice, considering both the effect of Gene × Diet and Gene × Sex × Diet, reporting ADONIS analysis and pairwise ANOSIM comparisons data including the sum of squares, mean squares, F value, R^2^, and respective p-values. *E4*-inulin mice experienced significant changes in β-diversity compared to its control (p = 0.001), with an overall diet effect among groups (ADONIS R^2^ = 0.436, F = 57.873, p = 0.001). *E3*-inulin mice showed a similar result, but a more pronounced impact was seen in *E4* mice from the figure. Further stratifying by sex, we found that both male and female mice had significant changes in β-diversity when fed with inulin, regardless of their *APOE* genotype. Figure 2b show these results for males and females; an overall diet (ADONIS R^2^ = 0.020, F = 2.817, p = 0.036) and sex effect (ADONIS R^2^ = 0.436, F = 60.175, p = 0.001) followed by pairwise comparisons: *E4*-M-control vs. *E4*-M-inulin p = 0.001, *E4*-F-control vs. *E4*-F-inulin p = 0.001, *E3*-M-control vs. *E3*-M-inulin p = 0.001, and *E3*-F-control vs. *E3*-F-inulin p = 0.003.

### Inulin increases the abundance of SCFAs-producing bacteria, primarily in acetate-producing bacteria in the *APOE4* male mice

The β-diversity changes led us to analyze further the present taxa to understand the taxonomic changes induced by inulin. Table 2 shows that inulin significantly increased the abundance of bacteria that produce SCFAs, including acetate, propionate, and butyrate^15–31^. A log_2_ FC represents the ratio of the normalized mean abundance of the gut microbiota in the *APOE3* and *APOE4* inulin groups relative to their control groups for comparison. A positive log_2_ fold change (FC) indicates that the relative abundance of a bacteria species increases in the inulin group compared to the controls, while a negative log_2_ FC indicates the opposite. Inulin increased the abundance of SCFAs-producing bacteria in *APOE4* mice compared to its control (Table 2). All the taxonomic changes with an FDR-corrected p-value (q) ≤ 0.05 were considered statistically significant in *APOE4* mice; specifically, 16 different microbial taxa were more abundant. Although inulin similarly boosted the population of SCFAs-producing bacteria in *APOE3* mice relative to its control group, the overall alteration in bacterial count (11) was less pronounced than in *APOE4* mice (Table 2).

**Table 2:** Gut microbiota taxonomy species changed by inulin in *APOE4* and *APOE3* mice (gene × diet) and *APOE4* and *APOE3* male and female mice (gene × sex × diet) compared to their controls, respectively.

SCFAs producers	Species	Gene × diet			Gene × sex × diet				
		Diet effect	*E4* $\frac{\mathrm{Inulin}}{\mathrm{Control}}$	*E3* $\frac{\mathrm{Inulin}}{\mathrm{Control}}$	Diet effect	*E4* Male $\frac{\mathrm{Inulin}}{\mathrm{Control}}$	*E4* Female $\frac{\mathrm{Inulin}}{\mathrm{Control}}$	*E3* Male $\frac{\mathrm{Inulin}}{\mathrm{Control}}$	*E3* Female $\frac{\mathrm{Inulin}}{\mathrm{Control}}$
		Q value	Log_2_ FC	Log_2_ FC	Q value	Log_2_ FC	Log_2_ FC	Log_2_ FC	Log_2_ FC
Acetate	*Bifidobacterium pseudolongum*	2.66E−10*	+ 9.88^†^	+ 12.05^†^	3.49E−09*	+ 12.95^†^	+ 8.02^†^	+ 16.04^†^	+ 11.27^†^
	*Bifidobacterium animalis*	3.84E−21*	+ 8.20^†^	+ 11.12^†^	8.06E−10*	+ 10.87^†^	+ 6.77^†^	+ 11.54^†^	+ 10.68^†^
	*Bifidobacterium choerinum*	8.01E−07*	+ 7.29^†^	+ 5.50^†^	1.80E−03*	+ 7.91^†^	+ 6.04^†^	+ 5.73^†^	+ 5.21^†^
	*Bifidobacterium bifidum*	0.09	+ 2.05^†^	+ 1.24	0.48	+ 2.68^†^	+ 1.37	+ 1.40	+ 1.00
	*Phocaeicola salanitronis*	5.22E−07*	+ 1.23^†^	+ 2.29^†^	0.03*	+ 2.47^†^	− 0.37	+ 2.67^†^	+ 1.81^†^
	*Parabacteroides goldsteinii*	0.71	+ 1.61^†^	− 0.22	0.23	+ 2.18^†^	+ 0.27	− 0.13	− 0.31
	*Bifidobacterium breve*	6.01E−07*	+ 1.69^†^	+ 3.07^†^	8.43E−05*	+ 1.93^†^	+ 1.24	+ 2.16^†^	+ 4.38^†^
Acetate and propionate	*Bacteroides uniformis*	5.97E−18*	+ 5.67^†^	+ 6.46^†^	1.83E−13*	+ 6.15^†^	+ 5.41^†^	+ 6.54^†^	+ 6.36^†^
	*Bacteroides thetaiotaomicron*	2.35E−05*	+ 4.47^†^	+ 3.06^†^	0.04*	+ 5.64^†^	+ 2.81^†^	+ 3.55^†^	+ 2.49^†^
	*Bacteroides xylanisolvens*	0.41	+ 1.11^†^	+ 0.43	0.23	+ 2.77^†^	− 1.07	+ 0.53	+ 0.33
	*Bacteroides fragilis*	2.53E−09*	+ 1.34^†^	+ 2.68^†^	1.13E−04*	+ 2.07^†^	+ 0.26	+ 2.62^†^	+ 2.82^†^
Butyrate	*Faecalibaculum rodentium*	7.58E−08*	+ 15.94^†^	+ 6.76^†^	4.87E−06*	+ 16.28^†^	+ 15.30^†^	+ 6.34^†^	+ 9.03^†^
	*Eubacterium limosum*	0.24	+ 4.31^†^	− 1.06	0.24	+ 3.98^†^	+ 4.82^†^	− 0.33	− 4.27^†^
	*Lachnoclostridium phocaeense*	0.11	+ 0.88^†^	+ 0.74	0.13	+ 0.85^†^	+ 0.92	− 0.16	+ 1.39
	*Anaerostipes hadrus*	0.88	+ 0.96	+ 0.09	0.94	+ 0.39	+ 2.07^†^	+ 0.99	− 1.16
All	*Odoribacter splanchnicus*	4.52E−03*	+ 0.99^†^	+ 1.53^†^	0.04*	+ 0.98^†^	+ 1.03^†^	+ 1.54	+ 1.58
	*Bacteroides ovatus*	2.51E−04*	+ 1.25^†^	+ 2.13^†^	5.12E−03*	+ 0.71	+ 1.75^†^	+ 3.45^†^	+ 1.14

Overall diet effect Q value, Log_2_ fold change (FC), and Q value for each species were calculated. Differences in SCFAs producers were reported.

*An overall diet effect Q value ≤ 0.05.

^†^A positive/negative value of log_2_ FC with a Q value ≤ 0.05 for the pairwise comparison denoted.

**Figure 3:**
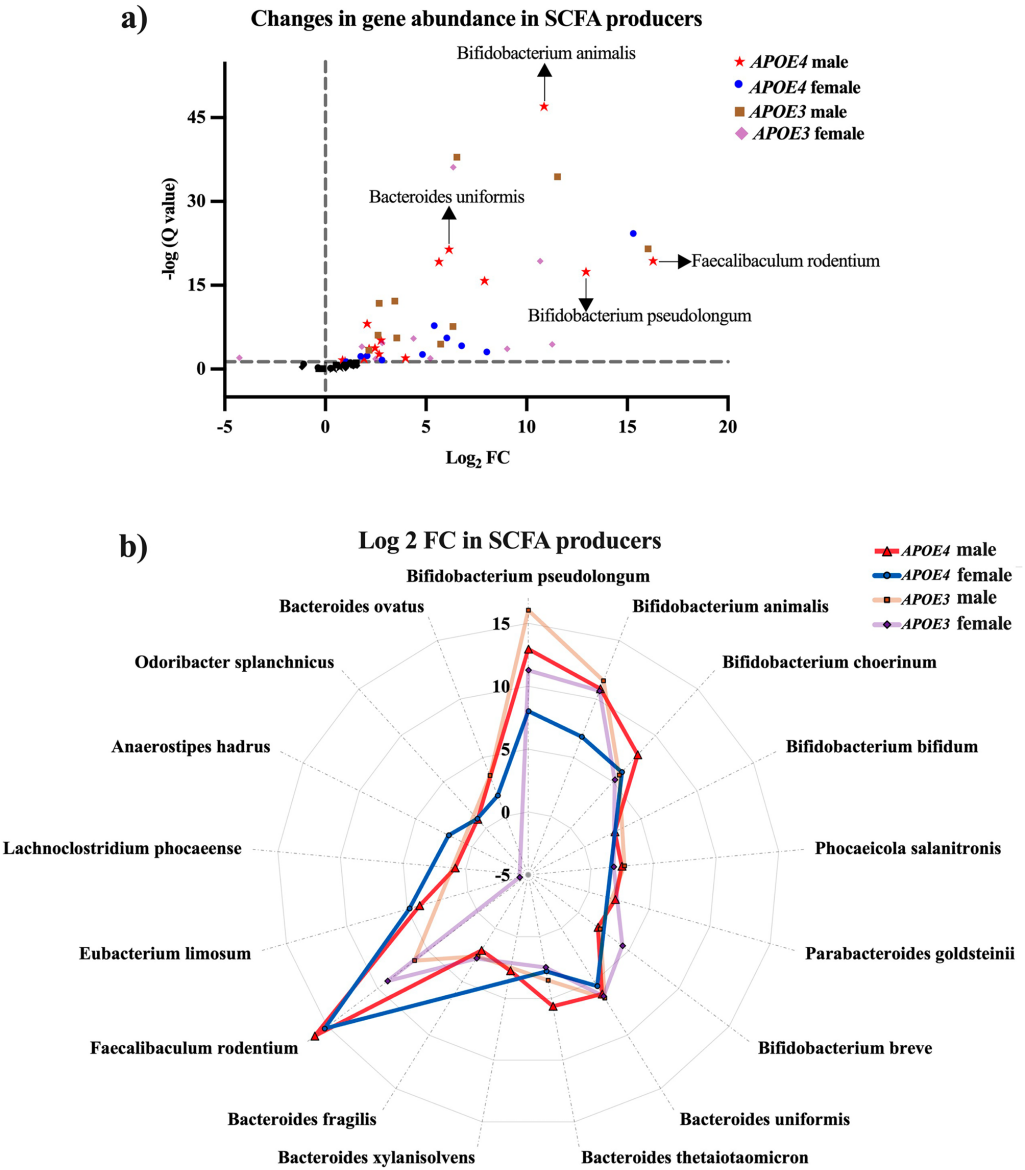
Fold changes (FCs) in the gene abundance of the gut SCFAs producers in *APOE*-inulin mice stratified by sex compared to their controls. (**a**) A one-sided volcano plot shows the log_2_ FC and -log (Q value) in gene abundance of SCFAs producers in the gut in *E4*-inulin and *E3*-inulin mice stratified by sex compared to their controls. Plots with a greater fold change and/or a more significant Q value in *E4*-M mice were indicated. (**b**) A radar chart reveals the log_2_ FC with a Q value ≤ 0.05 in gene abundance of SCFAs producers in the gut in *E4*-inulin and *APOE3*-inulin mice stratified by sex compared to their controls.

When stratified by sex, the one-sided volcano plot in Fig. 3a shows the log_2_ FC and − log (q value) in gene abundance in SCFAs-producing bacteria in all four groups receiving inulin compared to their controls. The radar chart in Fig. 3b illustrates the log_2_ FC in the 17 SCFAs-producing bacteria shown in Table 2. We found that *E4*-M-inulin vs. control mice showed more increases in SCFAs-producing bacteria compared to *E4*-F-inulin vs. control mice; 15 microbiota were increased in *E4*-M and 10 in *E4*-F. When we investigated specific gut microbiota, *E4*-M-inulin vs. control mice showed more increases in acetate-producing bacteria compared to *E4*-F-inulin vs. control mice; 12 microbiota were increased in *E4*-M, 7 in *E4*-F (Table 2, Fig. 3a,b). Inulin also modified the abundance of SCFAs-producing bacteria in *E3*-inulin vs. control group, but the extent of these changes was not as substantial as those observed in *APOE4* mice. Specifically, there was an increase in the number of the abundance of SCFAs-producing bacteria by 10 in *E3*-M mice and 9 in *E3*-F mice. Furthermore, the number of the abundance of acetate-producing bacteria saw an uptick by 9 in *E3*-M mice and 8 in *E3*-F mice (Table 2, Fig. 3a,b). Overall, *APOE4* mice and males had more changes than females, with *E4*-M showing the most significant changes.

### Inulin reduces the abundance of lactic acid bacteria (LAB) more significantly in the *APOE4* male mice while reducing *E. coli* more significantly in the *APOE4* female mice

**Figure 4:**
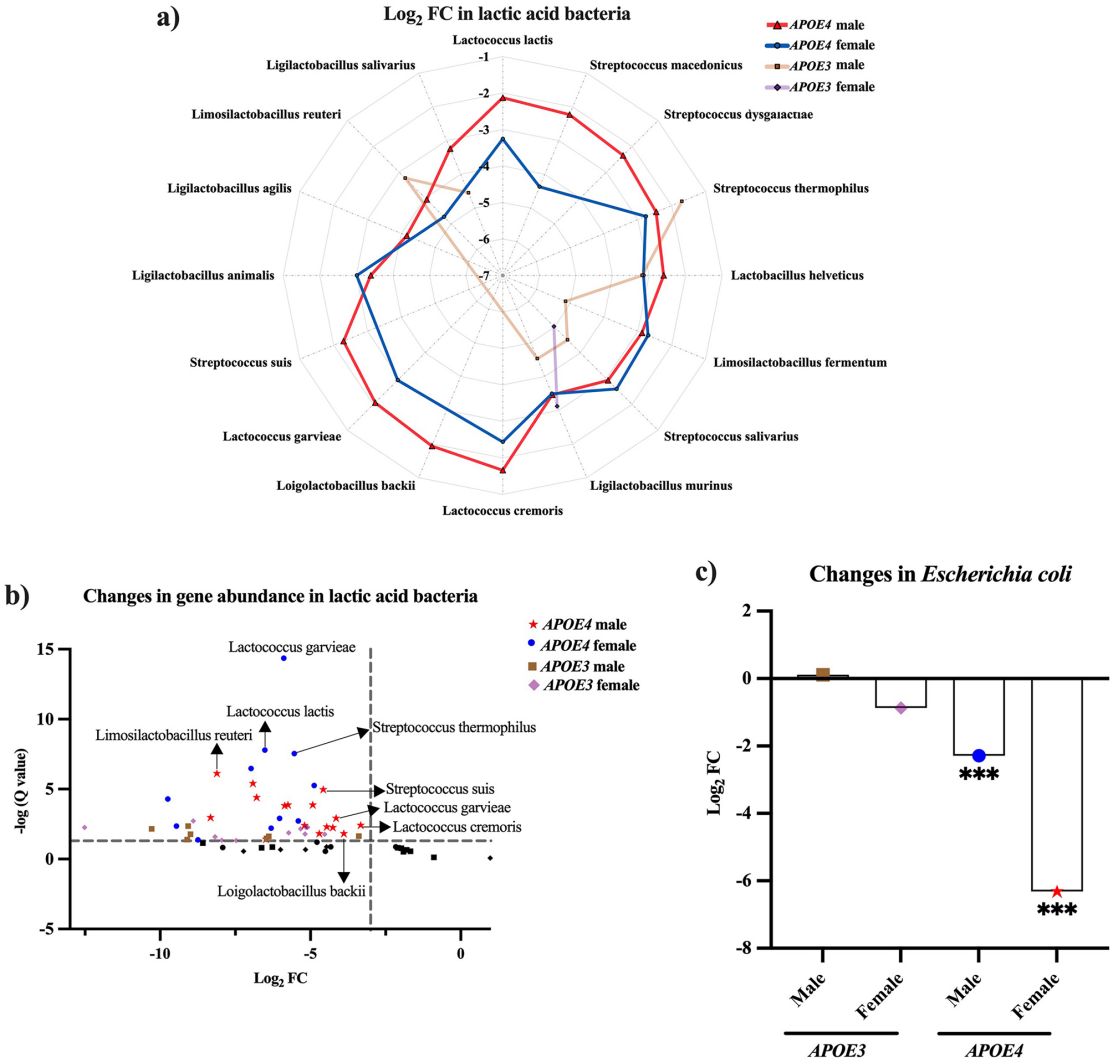
Fold changes (FCs) in the gene abundance of gut lactic acid bacteria (LAB) and *Escherichia coli* (*E. coli*) in *APOE*-inulin mice stratified by sex compared to their controls. (**a**) A radar chart revealed the log_2_ FC with a Q value ≤ 0.05 in gene abundance of LAB in the gut in *E4*-inulin and *E3*-inulin mice stratified by sex compared to their controls. (**b**) A one-sided volcano plot showed the log_2_ FC and -log (Q value) in gene abundance of LAB in the gut in *E4*-inulin and *E3*-inulin mice stratified by sex compared to their controls. Plots with a greater fold change and/or a more significant Q value in *E4*-M and *E4*-F were indicated. (**c**) Inulin decreased *E. coli* in *E4*-M and *E4*-F mice compared to their controls. *E3*-F-inulin and *E4*-M-inulin showed less *E. coli* than *E3*-M-inulin, while *E4*-F-inulin mice exhibited less *E. coli* than *E4*-M-inulin mice. ***Q ≤ 0.001. (**d**) A bar graph showed the Log_2_ FC in the gut *E. coli* in *E4*-inulin and *E3*-inulin mice stratified by sex compared to their controls. Inulin declined the abundance of *E. coli* in *E4*-M-inulin and *E4-*F-inulin mice compared to their controls. ***Q ≤ 0.001.

The differential analysis of the present taxa also revealed that inulin significantly reduced the abundance of LAB^32–43^ and the opportunistic pathogen *E. coli* (Table 3). All the taxa changes with a q ≤ 0.05 were considered statistical significance. Both *APOE4* and *APOE3* genotypes showed similar decreases in LAB and *E. coli* abundance. When further analyzed by sex, the radar chart in Fig. 4a illustrates the log_2_ FC, and the one-sided volcano plot in Fig. 4b depicts the log_2_ FC and q value in LAB among the four groups fed with inulin. We found that inulin led to a greater decrease in LAB in *E4*-M-inulin vs. control mice compared to *E4*-F-inulin vs. control mice, with a decrease of 16 species in *E4*-M and 11 in *E4*-F (Table 3, Fig. 4a,b). Inulin also decreased LAB in *E3*-inulin vs. control group, 7 in *E3*-M and 2 in *E3*-F (Table 3, Fig. 4a,b). Among those groups, *APOE4* mice overall showed a more significant number of changes than *APOE3* in the LAB decline regardless of sex.

**Table 3:** Gut microbiota lactic acid bacteria and *E. coli* species changed by inulin in *APOE4* and *APOE3* (gene × diet) and *APOE4* and *APOE3* male and female mice (gene × sex × diet) compared to their controls, respectively.

Category	Species	Gene × diet			Gene × sex × diet				
		Diet effect	*E4* $\frac{\mathrm{Inulin}}{\mathrm{Control}}$	*E3* $\frac{\mathrm{Inulin}}{\mathrm{Control}}$	Diet effect	*E4* Male $\frac{\mathrm{Inulin}}{\mathrm{Control}}$	*E4* Female $\frac{\mathrm{Inulin}}{\mathrm{Control}}$	*E3* Male $\frac{\mathrm{Inulin}}{\mathrm{Control}}$	*E3* Female $\frac{\mathrm{Inulin}}{\mathrm{Control}}$
		Q value	Log_2_ FC	Log_2_ FC	Q value	Log_2_ FC	Log_2_ FC	Log_2_ FC	Log_2_ FC
Lactic acid bacteria	*Lactococcus lactis*	0.04	− 2.47^†^	− 1.12^†^	0.03*	− 2.13^†^	− 3.26^†^	− 0.99	− 1.31
	*Streptococcus macedonicus*	0.17	− 2.53^†^	− 0.99^†^	0.07	− 2.23^†^	− 4.37^†^	− 0.84	− 1.21
	*Streptococcus dysgalactiae*	5.81E−04	− 2.22^†^	− 3.32^†^	0.04*	− 2.35^†^	− 2.25	− 3.31	− 2.70
	*Streptococcus thermophilus*	8.40E−04	− 2.59^†^	− 1.59^†^	0.02*	− 2.46^†^	− 2.77^†^	− 1.70^†^	− 1.40
	*Lactobacillus helveticus*	5.08E−04	− 2.72^†^	− 2.92^†^	0.04*	− 2.60^†^	− 3.15^†^	− 3.19^†^	− 1.79
	*Limosilactobacillus fermentum*	3.23E−06	− 2.82^†^	− 4.50^†^	3.51E−03*	− 2.88^†^	− 2.70^†^	− 5.14^†^	− 2.76
	*Streptococcus salivarius*	8.86E−06	− 3.19^†^	− 4.63^†^	9.94E−05*	− 2.93^†^	− 2.60^†^	− 4.50^†^	− 5.02^†^
	*Ligilactobacillus murinus*	3.13E−06	− 3.45^†^	− 4.40^†^	2.17E−03*	− 3.46^†^	− 3.49^†^	− 4.53^†^	− 3.12^†^
	*Lactococcus cremoris*	0.10	− 2.41^†^	− 1.10^†^	0.02*	− 1.66^†^	− 2.44^†^	− 0.90	− 1.44
	*Loigolactobacillus backii*	0.14	− 2.20^†^	− 1.13	0.07	− 1.94^†^	− 3.96	− 0.95	− 1.42
	*Lactococcus garvieae*	0.02	− 2.47^†^	− 1.13^†^	0.03*	− 2.07^†^	− 2.94^†^	− 1.05	− 1.21
	*Streptococcus suis*	0.21	− 1.83^†^	0.84	0.21	− 2.29^†^	− 1.08	− 0.45	+ 1.22
	*Ligilactobacillus animalis*	4.94E−05	− 3.27^†^	− 4.05^†^	0.02*	− 3.39^†^	− 3.01^†^	− 4.29	− 2.73
	*Ligilactobacillus agilis*	2.02E−03	− 3.61^†^	− 3.09	0.33	− 4.16^†^	− 2.39	− 3.13	− 1.62
	*Limosilactobacillus reuteri*	0.02	− 4.64^†^	− 2.30^†^	0.26	− 4.06^†^	− 4.73^†^	− 3.23^†^	− 0.58
	*Ligilactobacillus salivarius*	1.35E−03	− 2.62^†^	− 3.02^†^	0.27	− 3.24^†^	− 2.16	− 4.55^†^	− 1.82
Opportunistic pathogen	*Escherichia coli*	0.97	− 3.55^†^	− 0.02	0.13	− 2.29^†^	− 6.31^†^	+ 0.11	− 0.87

Overall diet effect Q value, Log_2_ fold change (FC), and Q value for each species were calculated. Differences in lactic acid bacteria and *E. coli* were reported.

*An overall diet effect Q value ≤ 0.05.

^†^A negative value of log_2_ FC with a Q value ≤ 0.05 for the pairwise comparison denoted.

In addition, the *E. coli* population was significantly reduced in *E4*-F-inulin (q = 2.00 × 10^–6^) and *E4*-M-inulin (q = 4.07 × 10^–3^) mice compared to their controls, with a greater fold change in *E4*-F mice (*E4*-F vs. *E4*-M: 12.62 folds vs. 4.58 folds, Table 3, Fig. 4c). The *E. coli* population did not change in *E3*-inulin mice compared to the controls when stratified by sex (Fig. 4c).

### Inulin differentially impacts the abundance of gut microbiota in SCFAs-related and metabolism-related kinases and pathways in both the *APOE4* and *APOE3* mice

**Figure 5:**
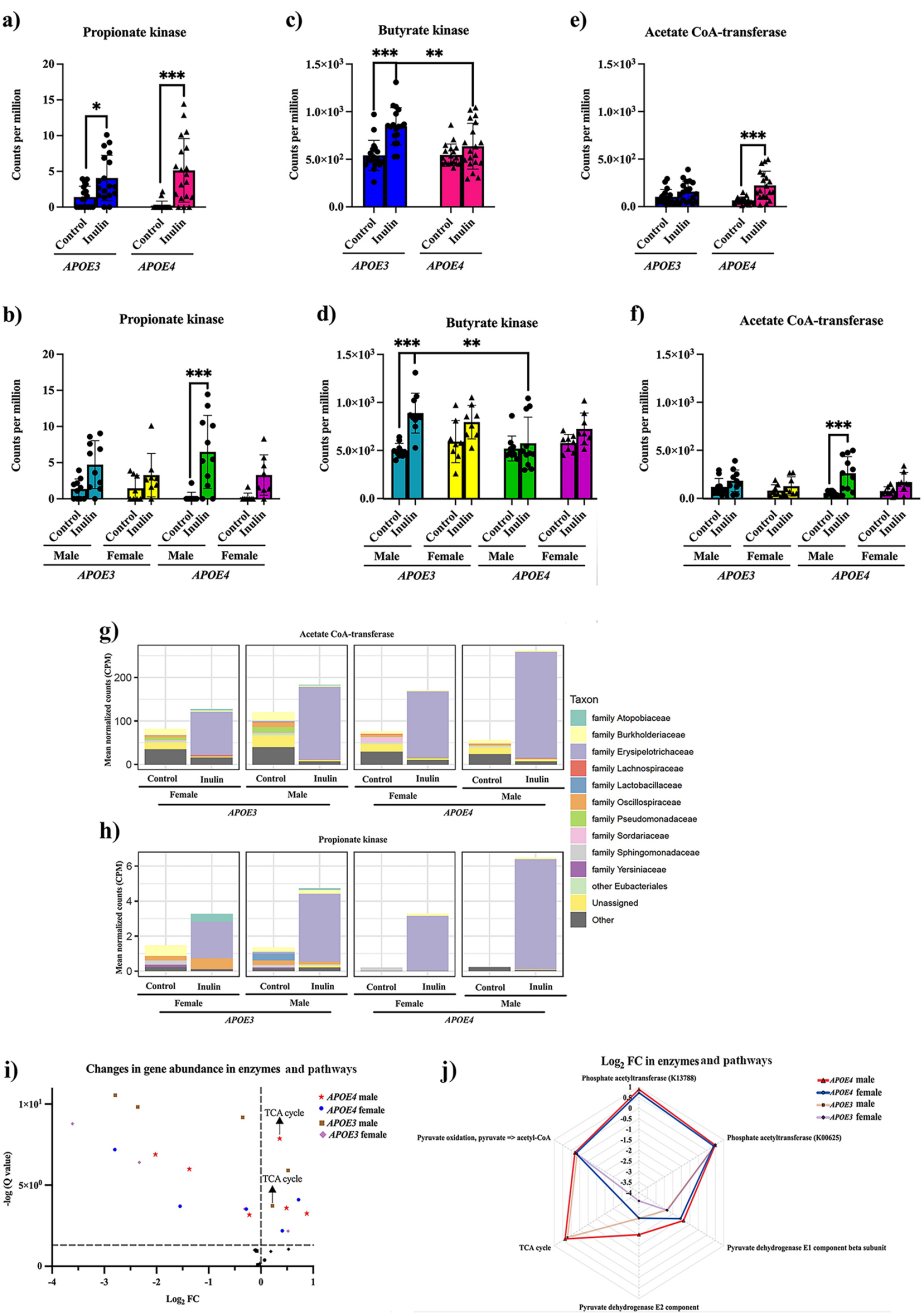
Changes in SCFAs-related kinases and fold changes (FCs) in functional gene abundance in *APOE3* and *APOE4* mice compared to their controls. Inulin increased the abundance of (**a**) propionate kinase and (**c**) butyrate kinase in *E3*-inulin mice and (**a**) propionate kinase and (**e**) acetate CoA-transferase in *E4*-inulin mice compared to their controls. When looking into the sex-dependent responses, inulin increased the abundance of (**d**) butyrate kinase in *E3*-M-inulin and (**b**) propionate kinase and (**f**) acetate CoA-transferase in *E4*-M-inulin mice compared to their controls. *p < 0.05. **p < 0.01. ***p < 0.001. Error bars show mean ± SD. Inulin increased the abundance of microbiota taxa that contributed to the abundance of (**g**) acetate CoA-transferase and (**h**) propionate kinase. (**i**) A one-sided volcano plot showed the log_2_ FC and -log (Q value) in gene abundance in metabolism- and inflammation-related kinases and pathways in *E4*-inulin and *E3*-inulin mice stratified by sex compared to their controls. Inulin changed the abundance of genes in the tricarboxylic acid (TCA) cycle in *E4*-M-inulin and *E3*-M-inulin mice compared to their controls. (**j**) A radar chart revealed the log_2_ FC with a Q value ≤ 0.05 in the gene abundance in metabolism- and inflammation-related kinases and pathways in *E4*-inulin and *E3*-inulin mice stratified by sex compared to their controls.

To gain further insight into how variations in the microbial taxa may affect metabolic pathways, we performed a 2-way ANOVA with experimental covariates (i.e., genotype, diet) followed by pairwise comparisons and a 3-way ANOVA with experimental covariates (i.e., genotype, diet, sex) followed by pairwise comparisons. We also performed a differential analysis of functional gene orthologs and higher level KEGG categories, i.e., pathways, modules, and BRITE levels, present. Results showed that inulin elevated the abundance of genes in propionate kinase in *APOE4* (p < 0.001) mice compared to their controls with an overall diet effect [F (1, 69) = 31.63, p < 0.001] among groups (Fig. 5a). When stratified by sex, the relative abundance of propionate kinase genes was increased in *E4*-M (p < 0.001; Fig. 5b). Inulin also increased the abundance of acetate CoA-transferase [F (1, 69) = 19.84, p < 0.001], but only in the *APOE4* group (p < 0.001, Fig. 5e) in male mice (p < 0.001; Fig. 5f). Investigation of the microbial taxa that contribute to these kinases revealed that the family *Erysipelotrichaceae* was a leading contributor. We found that inulin increased the abundance of family *Erysipelotrichaceae*, and that likely contributed to the change in acetate CoA transferase (Fig. 5g) and propionate kinase (Fig. 5h), especially in *E4*-M mice.

Table 4 shows the log_2_ FC in functional enzymes and pathways and their significance induced by inulin. We found enhancement of the abundance of the TCA cycle and suppression of glycolysis (glucose → pyruvate) and pyruvate oxidation (pyruvate → acetyl-CoA) in *E4*-inulin mice compared to its control. We observed that the abundance of the enzyme phosphate acetyltransferase (pta, K13788 in KEGG) and pta (K00625 in KEGG) increased in *E4*-inulin mice. We also observed a reduction in the abundance of the pyruvate dehydrogenase (PDH) E1 and E2 components in *E4*-inulin mice. Figure 5i,j display the log_2_ FC with q values and log_2_FC in functional enzymes and pathways found to be affected by inulin stratified by sex, as shown in Table 4. Inulin increased the abundance of orthologs in the TCA cycle in *E4*-M-inulin mice compared to its control.

**Table 4:** Differential analysis of functional gene abundance in *APOE4* and *APOE3* inulin mice (gene × diet) and *APOE4* and *APOE3* inulin male and female mice (gene × sex × diet) compared to their controls, respectively.

Category	Name	Gene × diet			Gene × sex × diet				
		Diet effect	*E4* $\frac{\mathrm{Inulin}}{\mathrm{Control}}$	*E3* $\frac{\mathrm{Inulin}}{\mathrm{Control}}$	Diet effect	*E4* Male $\frac{\mathrm{Inulin}}{\mathrm{Control}}$	*E4* Female $\frac{\mathrm{Inulin}}{\mathrm{Control}}$	*E3* Male $\frac{\mathrm{Inulin}}{\mathrm{Control}}$	*E3* Female $\frac{\mathrm{Inulin}}{\mathrm{Control}}$
		Q value	Log_2_ FC	Log_2_ FC	Q value	Log_2_ FC	Log_2_ FC	Log_2_ FC	Log_2_ FC
Kinases	Phosphate acetyltransferase (K13788)	0.38	+ 0.73^†^	+ 0.12	0.27	+ 0.88^†^	+ 0.72^†^	− 0.06	+ 0.53
	Phosphate acetyltransferase (K00625)	5.75E−09*	+ 0.38^†^	+ 0.46^†^	5.21E−05*	+ 0.49^†^	+ 0.41^†^	+ 0.52^†^	+ 0.52^†^
	Pyruvate dehydrogenase E1 component beta subunit	1.11E−13*	− 1.51^†^	− 2.39^†^	2.13E−10*	− 1.37^†^	− 1.55^†^	− 2.36^†^	− 2.33^†^
	Pyruvate dehydrogenase E2 component	4.19E−13*	− 2.33^†^	− 3.11^†^	7.06E−12*	− 2.02^†^	− 2.80^†^	− 2.79^†^	− 3.61^†^
Modules	TCA cycle	6.39E−05*	+ 0.21^†^	+ 0.18^†^	0.03*	+ 0.36^†^	+ 0.07	+ 0.22^†^	+ 0.19
	Glycolysis, glucose = > pyruvate	2.46E−05*	− 0.07^†^	− 0.13^†^	2.12E−04*	− 0.02	− 0.10	− 0.08	− 0.12
	Pyruvate oxidation, pyruvate = > acetyl-CoA	9.83E−14*	− 0.27^†^	− 0.37^†^	3.01E−09*	− 0.22^†^	− 0.28^†^	− 0.35^†^	− 0.31^†^

Overall diet effect Q value, Log_2_ fold change (FC), and Q value for each comparison were calculated. Differences in gene expression abundance in multiple kinases and modules were reported.

*An overall diet effect Q value ≤ 0.05.

^†^A positive/negative value of log_2_ FC with a Q value ≤ 0.05 for the pairwise comparison denoted.

Furthermore, inulin increased the abundance of propionate kinase in *APOE3* mice (p = 0.034; Fig. 5a), yet this effect was absent when we stratified them by sex (Fig. 5b). Inulin also induced the butyrate kinase with an overall diet effect F (1, 69) = 21.40, p < 0.001. This impact, however, was specific to *APOE3* mice (p < 0.001, Fig. 5c), particularly noticeable in *E3*-M mice (p < 0.001, Fig. 5d).

Inulin also stimulated an increase in the abundance of genes in the TCA cycle, pta (K00625 in KEGG), and PDH E1 and E2 components, while concurrently reducing the abundance of glycolysis and pyruvate oxidation in *APOE3* mice (Table 4). When stratified by sex, inulin heightened the abundance of the TCA cycle in *E3*-M mice relative to its control group (Fig. 5i,j, Table 4).

## Discussion

Our findings demonstrated that in *E4*-F and *E4*-M mice, the inulin diet elicited sex-dependent and distinct responses, with minimal impact on their food intake and body weight. *E4*-F control mice had increased α-diversity compared to the other groups, indicating gut microbiome dysbiosis. Previous studies have linked higher α-diversity to dysbiosis in conditions such as aging and stroke^44,45^. Interestingly, inulin was able to reduce and normalize the α-diversity of the *E4*-F mice to the level similar to other three groups. The finding aligns with previous studies demonstrating that inulin can reduce α-diversity in mice with *APOE4* genotype and with traumatic brain injury (TBI)^12,13,46^.

Another major finding from the *E4*-F mice was the reduced in the abundance of *E. coli.* Elevated *E. coli* levels have also been linked to diseases such as stroke, TBI, and AD^3,46,47^. In this study, inulin decreased the abundance of *E. coli*, which might reduce the Lipopolysaccharide (LPS)-induced inflammatory pathways^48–50^. Therefore, our results imply that by reducing *E. coli* and potentially alleviating inflammation-related pathways, inulin may mitigate neurodegeneration by regulating inflammatory mediators, especially in *E4*-F-inulin mice. The decline in the abundance of *E. coli* may be due to the induced abundance of SCFAs-producing bacteria in inulin-fed *APOE* mice as the gut microbial ecosystem shifted^51^.

In contrast to the *E4*-F, *E4*-M mice exhibited greater abundance of SCFA-producing bacteria and genes in metabolic changes, particularly in the SCFAs- and TCA-related pathways. Importantly, these effects related to diet and sex were less prominent in *APOE3* mice, indicating a significant role played by different *APOE* variants. Given that bioenergetic deficits are critical drivers of AD development^52,53^, these findings may provide insights into the higher susceptibility of *APOE4* females to AD, potentially resulting from inefficient energy production. Our results showed that inulin increases the population of bacteria that produce SCFAs, mainly acetate, after fermentation in the gut, with more dramatic changes shown in *E4*-M mice. Acetate has been demonstrated to have various potentially advantageous effects on the brain. For instance, studies show that acetate can lower microglial activation and inflammatory markers in neuro-inflammation models, alleviate thalamic neurodegeneration, and increase cerebral blood flow in patients with Alcohol Use Disorders^54–60^.

In the *E4*-M mice, we also observed an increased abundance of SCFAs-associated enzymes, including acetate CoA-transferase and propionate kinase. Notably, inulin enhanced the abundance of the family *Erysipelotrichaceae*, which was inconspicuous in control groups. This included the species *Faecalibaculum rodentium*, whose increase likely played a crucial role in elevating the abundance of acetate CoA-transferase and propionate kinase, especially in *E4*-M mice. These observations suggest a correlation between the increased presence of the family *Erysipelotrichaceae* and the abundance of SCFAs-related kinases. Further, we noted an increase in the abundance of the Pta enzyme in *E4*-M-inulin mice compared to its control. This may imply a potential conversion of acetate to acetyl-CoA via the Pta-Ack pathway^61^, potentially enhancing mitochondrial function^62^.

Regardless of *APOE* genotype or sex, we saw a decrease in the abundance of the PDH enzyme, which may lead to a decline in pyruvate oxidation^63^. The reduced abundance in pyruvate oxidation and glycolysis may increase acetyl-CoA production, which may further increase the abundance in the TCA cycle^64–69^. This shift in energy sources within the gut in our results unveils that inulin may increase glucose availability for the brain and neurons by serving as a source of acetyl-CoA, thereby promoting proper neural function. The family *Erysipelotrichaceae* has been linked to inflammation following a high-fat or Western diet^70^. Interestingly, it has been found that *Faecalibaculum rodentium*, the species within this family, produces SCFAs and offers protective effects against cancer^28^. This aligns with our prior observation that inulin increases SCFAs-producing bacteria, potentially augmenting SCFAs production and improving mitochondrial function. These findings underscore the importance of studying specific species within the gut microbiota for their distinct roles.

Another key finding from the present study is the reduced LAB regardless of *APOE* genotype or sex. The increased SCFAs production due to inulin likely decreased the abundance of LAB, again, as the gut microbial ecosystem altered^51^. The decrease in LAB abundance may prevent excessive lactate production. The decreased LAB levels in *APOE4* mice may point to a reduction in lactate synthesis in the gut, which lowers the risk of disease pathology, including inflammation and cancer^71^.

*APOE4* is the strongest genetic risk factor for AD, and currently, there are no effective therapeutics to restore brain functionality after clinical symptoms have manifested. Accumulating evidence has shown that neurologically and systemically, metabolism may play a more critical role than amyloid beta plaques and tau tangles in AD progression^52^. In line with the findings in the present study, it has been shown that *APOE3* and *APOE4* carriers develop AD through different metabolic pathways^72^; therefore, it will be critical for future studies to identify precision nutrition approaches that tailored to different *APOE* variants to mitigate AD risk. In our prior work, we have demonstrated that inulin reduces neuroinflammatory gene expression and boosts SCFAs production in *APOE4* mice, which mitigates their risk for AD development^12,13^. Our research suggest that inulin could be a preventative intervention. However, evidence is currently sparse regarding inulin's efficacy as a treatment after AD symptoms have manifested. This is a vital avenue for future exploration.

In summary, we investigated the effects of inulin on the potential gut microbial metabolism and revealed that treatment effectiveness was associated with sex in *APOE4* carrier by analyzing the changes in gut microbiota and abundance of microbial metabolism in fecal samples. Our findings highlight the importance of considering sex in *APOE4* carriers when exploring the link between diet, gut microbiome, and AD risk mitigation. It would be important to explore the development of gender-specific treatments, like acetate-producing bacteria supplementation for *APOE4* females. It would also be beneficial to develop interventions tailored to different *APOE* variants. The findings from the study may provide insight for future precision nutrition applications to mitigate AD risk via gut-brain axis.

## Methods

### Animals and study design

We used a C57BL/6 mouse model with human-targeted replacement *APOE* (*ε4* in homozygous *APOE4* mice and *ε3* in homozygous *APOE3* mice) from Taconic (*APOE4* model number: 1549-M and 1549-F and *APOE3* model number: 1548-M and 1548-F). The mice were categorized into groups based on genotype and diet: *E4*-control (n = 18), *E4*-inulin (n = 19), *E3*-control (n = 17), and *E3*-inulin (n = 18). Additionally, groups were determined based on genotype, diet, and sex, resulting in the following categories: *E4*-M-control (n = 10), *E4*-F-control (n = 8), *E4*-M-inulin (n = 11), *E4*-F-inulin (n = 8), *E3*-M-control (n = 10), *E3*-F-control (n = 7), *E3*-M-inulin (n = 10), and *E3*-F-inulin (n = 8).

We fed mice a prebiotic inulin or control diet at four months and fed them for 16 weeks. Both diets were provided by TestDiet (control diet: 9GLK and inulin diet: 9GLL). The prebiotic inulin diet contained 8% fiber from inulin, and the control diet contained 8% fiber from cellulose, as we previously reported^12,13,73^. We fed the mice 8% of inulin because it has been shown that 8% of inulin increased cecal contents, produced more SCFA, and increased the amount of bacterial enzymes in the cecum compared to 4% of inulin^74^. Also, human studies showed that 8% of fiber (40 g of fiber per day) was considered the maximum amount for the western people to tolerate without side effects^75^. The detailed diet composition was provided in Table 5. We individually housed each mouse to avoid feces exchange with ad libitum access to food and water. We recorded the food intake and body weight biweekly. We collected the fecal samples when the mice reached eight months of age. The Institutional Animal Care and Use Committee (IACUC) at the University of Kentucky (UK) approved this study.

**Table 5:** Diet composition.

Diet	Prebiotic inulin diet	Control cellulose diet
Protein %	18.2	18.2
Carbohydrate %	59.1 (w/o inulin contribution)	60.2 (w/o cellulose contribution)
Fat %	7.1	7.1
Fiber %	8.0 (inulin)	8.0 (cellulose)
Energy (kcal/g)	4.08	3.78

### Fecal sample collection and gut microbiome analysis

Fecal samples were collected and frozen at − 80 °C until DNA extraction. Genomic DNA from feces was extracted using a ZymoBIOMICS DNA Mini Kit according to the manufacturer’s instructions. Shotgun metagenome libraries were prepared from fecal DNA using an Illumina DNA Prep kit according to the manufacturer’s instructions. The final library pool was sequenced on an Illumina NovaSeq6000 instrument using an S4 flow cell with paired-end 2 × 150 sequencing reads. Library preparation was performed at the Genomics and Microbiome Core Facility (GMCF) at Rush University, and sequencing was performed at the W.M. Keck Center for Comparative and Functional Genomics at the University of Illinois, Urbana-Champaign (UIUC). For taxonomic annotation, raw reads were mapped to the NCBI nucleotide database by Centrifuge for taxonomic annotation^76^. The least common ancestor algorithm was used for taxonomic annotations for each read. The annotations were then summarized across all reads to create counts per taxon. For functional gene annotations, raw reads were mapped to the Swissprot protein database using DIAMOND^77,78^. Gene orthologs annotations were then assigned using the consensus of aligned references and then summarized across all reads to create counts per ortholog for each sample. Higher-level summaries of orthologous functions are created using KEGG BRITE hierarchical annotations^79^. Raw counts were normalized to percentages for relative abundance.

### Statistics and reproducibility

#### Sample size determination

A sample size was calculated for an anticipated 86% power to detect at least a 60% difference in comparing *APOE4*-control and *APOE4*-inulin, assuming a common standard deviation of 20 using a 2-way ANOVA considering all pairwise post-hoc comparisons (α = 0.05, β = 0.20).

#### Food intake and body weight

We monitored both food intake and body weight of the mice biweekly over a span of 16 weeks. To analyze the changes in food intake over this period and the body weight at its conclusion, we used a 2-way ANOVA with experimental covariates (i.e., genotype, diet) followed by pairwise comparisons and a 3-way ANOVA with experimental covariates (i.e., genotype, diet, sex) followed by pairwise comparisons.

#### Beta diversity/dissimilarity analyses

Before analysis, the normalized data were square root transformed. Using the vegan library, the Bray–Curtis index was calculated with default parameters in R (v3.6.2, https://www.r-project.org/)^80^. The resulting dissimilarity index was modeled and tested using the ADONIS test for significance with the sample covariates. Additional comparisons of the individual covariates were performed using ANSOIM. Plots were generated in R using the ggplot2 library^81^. A p-value less than 0.05 was considered statistically significant.

#### Alpha diversity analyses

Before analysis, the data were filtered as described above and rarefied to 1,000,000 counts per sample depth. The vegan library calculated the Shannon index with default parameters in R^80^. The resulting Shannon indices were then modeled with the sample covariates using a generalized linear model (GLM) assuming a Gaussian distribution. F-test was used to test the significance of the model (ANOVA). Post-hoc, pairwise tests were performed using the Mann–Whitney test. Plots were generated using the ggplot2 library in R^81^. A p-value less than 0.05 was considered statistically significant.

#### Differential relative abundance analysis of microbial taxonomy

Differential analyses of bacterial taxonomy compared with experimental covariates (i.e., genotype, diet, sex) were performed using the software package edgeR on raw sequence count^82^. The data were filtered to those taxa that were annotated as Bacteria but not annotated as chloroplast or mitochondria in origin, and removing taxa removed any taxon that had less than 1000 total counts across all samples and were present in less than 20% of the samples. Data were normalized as counts per million. Then normalized data were fit using a negative binomial GLM using experimental covariates (i.e., diet, sex, and genotype), and statistical tests were performed using a likelihood ratio test. A multi-factor comparison and repeated measures for the gut microbiome differential analysis were performed. Log_2_ fold-change (FC) and Q-value (false discovery rate-corrected P-value) for each taxon were reported. The Q value was calculated using the Benjamini–Hochberg false discovery rate (FDR) correction^83^. Significant taxa were determined based on an FDR threshold of 5% (0.05). The radar charts of log_2_ FC of SCFAs producers, LAB, and *E. coli* were created by Microsoft Office Excel (2022, https://www.microsoft.com/en-in/microsoft-365/excel). The one-sided volcano plots of log_2_ FC of SCFAs producers and LAB were created by GraphPad Prism 9 (https://www.graphpad.com/scientific-software/prism/).

#### Differential relative abundance analysis of microbial functional gene and pathway

Before analyses, data were filtered to only include features with at least 100 counts across all samples and present in at least 30% of the samples for kinase level (KOs) analyses. For module and pathway analyses, data were filtered only to include a feature with at least 1000 counts across all samples and present in at least 30% of the samples. Differential analyses of functional genes compared with experimental covariates (i.e., genotype, diet, sex) were performed using GLM and various pairwise comparisons. Log_2_ FC and Q-value for each KO and pathway were reported. Significant taxa were determined based on an FDR threshold of 5% (0.05). For changes in SCFAs-related kinases gene abundance, we performed a 2-way ANOVA with experimental covariates (i.e., genotype, diet) followed by pairwise comparisons and a 3-way ANOVA with experimental covariates (i.e., genotype, diet, sex) followed by pairwise comparisons. A p-value less than 0.05 was considered statistically significant. To investigate the microbiota that contributed to the abundance of SCFAs-related kinases abundance, we determined the taxonomic annotations and total read counts for each KO, computed the fractional read counts for each taxon for a particular KO, and obtained normalized read counts for each taxon for each taxon for the particular KO by multiplying the computed fractional read counts by the previously normalized counts for the KO. The bar charts were generated in R. The radar chart of log_2_ FC of metabolism- and inflammation-related kinases and pathways was created by Microsoft Office Excel (2022, https://www.microsoft.com/en-in/microsoft-365/excel). The one-sided volcano plot of log_2_ FC of metabolism- and inflammation-related kinases and pathways was created by GraphPad Prism 9. The schematic diagram was created by Microsoft PowerPoint for Microsoft 365 MSO (v2210, https://www.microsoft.com/en-us/microsoft-365/powerpoint).

## Data Availability

The gene amplicon sequence data generated for this study have been submitted to the NCBI Bio-Project database (PRJNA880558). The datasets generated during and/or analyzed during the current study are available from the corresponding author upon reasonable request.
